# Religion, Geography, and Risky Sexual Behaviors Among International Immigrants Living in China: Cross-Sectional Study

**DOI:** 10.2196/44616

**Published:** 2024-06-28

**Authors:** Yuyin Zhou, Feng Cheng, Junfang Xu

**Affiliations:** ^1^Department of Pharmacy, Second Affiliated Hospital, School of Public Health, Zhejiang University School of Medicine, Hangzhou, China; 2 Vanke School of Public Health, Tsinghua University, Beijing, China; 3 Institute for Healthy China, Tsinghua University, Beijing, China

**Keywords:** religion, geography, risky sexual behaviors, international immigrants, China, sexual behavior, immigrant

## Abstract

**Background:**

Behavioral differences exist between countries, regions, and religions. With rapid development in recent decades, an increasing number of international immigrants from different regions with different religions have settled in China. The degrees to which sexual behaviors—particularly risky sexual behaviors—differ by religion and geographical areas are not known.

**Objective:**

We aim to estimate the associations of religion and geographical areas with sexual behaviors of international immigrants and provide evidence for promoting the sexual health of international immigrants.

**Methods:**

A cross-sectional study was conducted via the internet with a snowball sampling method among international immigrants in China. In our study, risky sexual behaviors included having multiple sexual partners and engaging in unprotected sex. Descriptive analysis was used to analyze the basic characteristics of international immigrants as well as their sexual behaviors, religious affiliations, and geographical regions of origin. Multivariate binary logistic regression analyses with multiplicative and additive interactions were used to identify aspects of religion and geography that were associated with risky sexual behaviors among international immigrants.

**Results:**

A total of 1433 international immigrants were included in the study. South Americans and nonreligious immigrants were more likely to engage in risky sexual behaviors, and Asian and Buddhist immigrants were less likely to engage in risky sexual behaviors. The majority of the Muslims had sexually transmitted infection and HIV testing experiences; however, Muslims had a low willingness to do these tests in the future. The multivariate analysis showed that Muslim (adjusted odds ratio [AOR] 0.453, 95% CI 0.228‐0.897), Hindu (AOR 0.280, 95% CI 0.082‐0.961), and Buddhist (AOR 0.097, 95% CI 0.012‐0.811) immigrants were less likely to report engaging in unprotected sexual behaviors. Buddhist immigrants (AOR 0.292, 95% CI 0.086‐0.990) were also less likely to have multiple sexual partners. With regard to geography, compared to Asians, South Americans (AOR 2.642, 95% CI 1.034‐6.755), Europeans (AOR 2.310, 95% CI 1.022‐5.221), and North Africans (AOR 3.524, 95% CI 1.104‐11.248) had a higher probability of having multiple sexual partners.

**Conclusions:**

The rates of risky sexual behaviors among international immigrants living in China differed depending on their religions and geographical areas of origin. South Americans and nonreligious immigrants were more likely to engage in risky sexual behaviors. It is necessary to promote measures, including HIV self-testing, pre-exposure prophylaxis implementation, and targeted sexual health education, among international immigrants in China.

## Introduction

Culture is a complicated phenomenon, and there are important cultural differences between different countries, regions, and religions. International immigrants undergo an acculturation process, which influences how well they can live a new life in their new country and may result in differences in individual sexual behaviors [1]. Traditionally, in most countries, religious individuals have conservative attitudes toward sexual behavior [2,3]. For example, Christianity prohibits adolescents and unmarried ones from having sexual intercourse [4,5]. Similarly, Muslims are only permitted to have sex within marriage [6]. Some international immigrants have strong and deep-rooted religious beliefs derived from their motherland, and some of these beliefs may influence their sexual behaviors. Consequently, some international immigrants may be less likely to engage in risky sexual behaviors compared to local nonreligious people. However, some studies have found opposite results, indicating that religion may be a risk factor for engaging in risky sexual behaviors. Many world religions promote doctrines that include negative views toward homosexual behavior, multiple sexual partners, and premarital sexual behaviors. These doctrines may cause sexual guilt in individuals engaging in nonmonogamous sexual activity, which is associated with reduced contraceptive use [7,8]. In addition, a meta-analysis [9] focused on youths identified that religion was a protective factor for age at sexual debut and the number of sexual partners but had no association with contraceptive use. Individuals’ geographical region of origin also has associations with their sexual behaviors. It was reported that polygamy was one of the main factors contributing to risky sexual behaviors and HIV infection among Haitian immigrant women living in America [10]. Additionally, single Latina women were likely to have multiple sexual partners in America [11]. Moreover, Huang [12] indicated that the migration process, especially from middle- and low-income countries to high-income countries may increase the possibility of risky sexual behaviors.

With rapid development in recent decades, an increasing number of international immigrants have settled in China. The National Bureau of Statistics reported that approximately 845,697 international immigrants lived in China in 2020, representing an increase of 251,865 compared to 2010 [13,14]. However, international immigrants tend to be exposed to sexual vulnerability [15] for several reasons. The totally new living environment, including historical and cultural differences, huge language barriers, and lack of solid social networks among international immigrants can lead to social isolation, so they may engage in risky sexual behaviors to relax and spend spare time [16]. Meanwhile, the lack of community supervision brings sexual freedom among these immigrants, which may also increase their risky sexual behaviors as they seek mental and physical pleasure [17]. For instance, a previous study found that risky sexual behaviors among homosexual/bisexual male migrants from Central and Eastern Europe living in London had been significantly influenced by the process of migration. Specifically, disentangled from the conventional frameworks of social regulation prevalent in their countries of origin and with expanded access to gay venues in London led to a notable escalation in their sexual engagement [18]. Therefore, it has also been reported that migrants are an at-risk population vulnerable to contracting sexually transmitted infections (STIs) or HIV [19-21]. However, rates of engagement in risky sexual behaviors may differ by religion and geographical region of origin. This study investigated the associations of religion and geographical region of origin with the sexual behaviors of international immigrants living in China. Findings from this study can generate evidence to assist the promotion of sexual health and implement programs for target populations at high risk of sexual behaviors.

## Methods

### Participants

A cross-sectional study was conducted via the internet during the COVID-19 pandemic between January 2021 and September 2021. Considering the cultural differences and sensibility of sexual issues, the respondent-driven sampling method was used to access international immigrants living in China. First, 3‐6 international immigrants with plenty of social contacts living in different cities in China were chosen as the initial seed participants, and these seed participants had different occupations, ages, geographical regions of origin, and genders. After introducing the inclusion criteria of participants to every seed participant, they were required to recommend 1‐3 international immigrants. If a seed participant did not recruit any other participants within 2 weeks or recruited fewer than 5 participants over 2 months, a new seed participant corresponding to its sociodemographic characteristics would be developed to ensure survey continuity and seed diversity.

Guangdong province, Zhejiang province, and Beijing were chosen as the study sites. We selected these sites because the Seventh Population Census of China in 2022 showed that these provinces or municipalities were included in the top 10 provinces or municipalities in terms of the size of international immigrants in China [22]. Guangdong is the province with the largest number of international immigrants in China, with more than 410,000 international immigrants residing there. Guangdong’s open policy and diverse social environment provide good living and working conditions for international immigrants. Zhejiang Province has a developed economy, convenient transportation, and many private enterprises, with more than 46,000 international immigrants living there. Moreover, Yiwu in Zhejiang province is one of the largest commodity trading cities, attracting nearly half a million international immigrants every year [23]. Beijing is the capital and the political, economic, and cultural center of China, with over 62,000 international immigrants. Foreign embassies, offices of major foreign companies in China, commercial and trade organizations, and foreign students are all gathered in Beijing.

The inclusion criteria of the participants were the following: (1) their home country is not China, but they currently live in China for more than 3 months; (2) aged ≥18 years; (3) able to read in English; and (4) willing to participate in the study. The exclusion criteria were the following: (1) short stay in China for business, travel, and other purposes and (2) failure to pass the “attention check” in the questionnaire, which was set to identify the careless respondents and improve the data quality.

PASS 2021 (NCSS LLC) software was used to calculate the sample size. Previous studies [24,25] showed that the prevalence of HIV among international immigrants was 1.70%‐62.0%, so the prevalence of HIV was selected as 31.85% in this study with an α error set at .05, implying a 3% margin of error. To ensure the validity of the questionnaire, we increased the sample size by 20%, which required 1150 participants. Finally, 1460 participants were surveyed, and 1433 eligible participants were included in this study.

### Data Collection

All participants completed a web-based questionnaire in English, which included sociodemographic information (eg, home country, gender, age, marital status, religious beliefs, and education level), sexual behavior–related items, and the willingness to undergo testing.

The geographical regions of origin included sub-Saharan Africa, North America, South America, Europe, North Africa, Asia, and “others.” The religious beliefs included Christianity, Islam, Hinduism, Buddhism, and “others,” and a group of participants who identified as nonreligious were also included.

Sexual behavior–related items included whether one had participated in sexual behavior before, sexual orientation, number of sexual partners, and the frequency of condom use. Risky sexual behaviors included having multiple sexual partners and unprotected sexual behaviors. Multiple sexual partners referred to engaging in sexual behaviors with at least 1 other person in addition to their spouse or stable partner within the last year. Unprotected sexual behaviors referred to ever having sex without a condom. The questions regarding sexual behaviors were designed based on the guidelines for the prevention of HIV/AIDS issued by the China Center for Disease Control and our previous studies [26-28].

The willingness to undergo testing included STI/HIV test experiences (ie, whether they had had an HIV test in the past), perceived risk of being infected with STIs/HIV, and willingness for STI/HIV testing in the future.

### Statistical Analysis

The basic characteristics of international immigrants, and their sexual behaviors, religious beliefs, and geographical regions of origin were analyzed using descriptive statistics with frequencies and percentages. Multivariate binary logistic regression analyses were used to identify religion-related and geographical origin-related factors significantly associated with risky sexual behaviors among international immigrants. Additionally, in logistic regression analysis, we took having multiple sexual partners and unprotected sexual behaviors as the dependent variables. We considered age, gender, marital status, education level, employment status, annual disposable income, STI/HIV testing experience, perceived risk of being infected with HIV, and willingness for STI/HIV testing in the future variables as covariates for conducting a multiplicative and additive interaction analysis to further explore the interaction between religion and geography. The statistical software R (version 4.2; R Foundation for Statistical Computing) and SPSS (version 23.0; IBM Corp) were used to analyze all data, and *P*<.05 was considered to indicate statistical significance.

### Ethical Considerations

The study protocol and consent procedure were approved by the Ethics Review Committee, School of Public Health, Zhejiang University (#2019‐064). Informed consent information was provided before the questions; participants had the option to exit the survey after reading the informed consent information or to provide consent to continue. The confidentiality of individuals was properly protected in the management of the investigation and the processing of data.

## Results

### Basic Characteristics of International Immigrants

As shown in Table 1, a total of 1433 international immigrants living in China were included in the study, with the mean age of 24.97 (SD 4.57) years. The majority were male (n=973, 67.90%), and more than half were unmarried (n=1263, 88.14%). Participants who had completed over 12 years of education (n=831, 57.99%) and their annual disposable income was at most 50,000 yuan (n=1040, 72.58%) accounted for the largest percentage in all groups. The participants’ religions included Christianity (n=641, 44.73%), Islam (n=565, 39.43%), Hinduism (n=73, 5.09%), Buddhism (n=47, 3.28%), and others (n=11, 0.77%), and 95 (6.63%) had no religion. The geographical regions of origin included sub-Sahara Africa (n=665, 46.41%), North America (n=44, 3.07%), South America (n=23, 1.61%), Europe (n=40, 2.79%), North Africa (n=14, 0.98%), Asia (n=610, 42.57%), and others (n=6, 0.42%). Moreover, 37.68% (n=540) of the participants had risky sexual behaviors before.

**Table 1.** The basic characteristics of international immigrants in China (N=1433).

Variable		Values
Age (years), mean (SD)		24.97 (4.57)
**Age (years), n (%)**		
	≤20	90 (6.28)
	21‐30	903 (63.01)
	31‐40	306 (21.35)
	>40	134 (9.35)
**Gender, n (%)**		
	Male	973 (67.90)
	Female	460 (32.10)
**Marital status, n (%)**		
	Unmarried	1263 (88.14)
	Married	110 (7.68)
	Widowed	4 (0.28)
	Divorced	9 (0.63)
	Others	47 (3.28)
**Education level, n (%)**		
	Illiteracy	49 (3.42)
	1‐5 years	299 (20.87)
	6‐10 years	97 (6.77)
	11‐12 years	157 (10.96)
	>12 years	831 (57.99)
**Employment status, n (%)**		
	Employed	231 (16.12)
	Unemployed	1202 (83.88)
**Annual disposable income (￥^a^), n (%)**		
	≤50,000	1040 (72.58)
	50,001‐100,000	212 (14.79)
	100,001‐150,000	79 (5.51)
	>150,000	102 (7.12)
**Religion, n (%)**		
	Christianity	641 (44.73)
	Islam	565 (39.43)
	Hinduism	73 (5.09)
	Buddhism	47 (3.28)
	Others	11 (0.77)
	No religion	95 (6.63)
**Geography, n (%)**		
	Sub-Saharan Africa	665 (46.41)
	North America	44 (3.07)
	South America	23 (1.61)
	Europe	40 (2.79)
	North Africa	14 (0.98)
	Asia	610 (42.57)
	Others	6 (0.42)
**Whether they had risky sexual behaviors before, n (%)**		
	Yes	540 (37.68)
	No	893 (62.32)

a A currency exchange rate of Chinese Yuan ￥1=US $0.14 is applicable.

### Sexual Behaviors Among International Immigrants by Religion and Geography

Table 2 shows the distributions of risky sexual behaviors among international immigrants by religion. The rates of participants with multiple sexual partners were as follows: 30.53% (n=29) among nonreligious individuals, 27.93% (n=179) among Christians, 16.64% (n=94) among Muslims, 16.64% (n=12) among Hindus, and 8.51% (n=4) among Buddhists. The rates of unprotected sexual behaviors were as follows: 22.11% (n=21) among participants without religion, 17.63% (n=113) among Christians, 8.32% (n=47) among Muslims, 5.48% (n=4) among Hindus, and 4.26% (n=2) among Buddhists. Table 3 shows the distributions of risky sexual behaviors among international immigrants by geographical region of origin. The rates of participants who reported having multiple sexual partners were as follows: 52.17% (n=12) from South America, 35.71% (n=5) from North Africa, 32.50% (n=13) from Europe, 22.73% (n=10) from North America, 27.22% (n=181) from sub-Saharan Africa, and 15.74% (n=96) from Asia. Engaging in unprotected sexual behaviors was reported as follows: 30.43% (n=7) in those from South America, 20% (n=8) in those from Europe, 16.99% (n=113) in those from sub-Saharan Africa, 15.91% (n=7) in those from North America, 14.29% (n=2) in those from North Africa, and 7.87% (n=48) in those from Asia.

**Table 2.** Risky sexual behaviors of international immigrants by religion (N=1432^a^).

Religion	Multiple sexual partners, n (%)	Unprotected sexual behaviors, n (%)
Christianity (n=641)	179 (27.93)	113 (17.63)
Islam (n=565)	94 (16.64)	47 (8.32)
Hinduism (n=73)	12 (16.64)	4 (5.48)
Buddhism (n=47)	4 (8.51)	2 (4.26)
Others (n=11)	3 (27.27)	2 (18.18)
No religion (n=95)	29 (30.53)	21 (22.11)

a One person was missed in the reporting.

**Table 3.** Risky sexual behaviors of international immigrants by geography.

Geography	Multiple sexual partners, n (%)	Unprotected sexual behaviors, n (%)
Sub-Saharan Africa (n=665)	181 (27.22)	113 (16.99)
North America (n=44)	10 (22.73)	7 (15.91)
South America (n=23)	12 (52.17)	7 (30.43)
Europe (n=40)	13 (32.50)	8 (20)
North Africa (n=14)	5 (35.71)	2 (14.29)
Asia (n=610)	96 (15.74)	48 (7.87)
Others (n=6)	2 (33.33)	2 (33.33)

### The Willingness to Undergo Testing Among International Immigrants by Religion and Geography

Table 4 shows that 378 (58.97%) Christians, 469 (83.01%) Muslims, 58 (79.45%) Hindus, 3 (76.60%) Buddhists, and 65 (68.42%) nonreligious people had STIs/HIV test experiences. There were 348 (61.59%) Muslims and 46 (63.01%) Hindus who believed that it was impossible for them to be infected with STIs/HIV, which accounted for the largest percentages. As for the willingness to undergo STIs/HIV testing in the future, the willingness of Muslims was relatively low and the willingness of Christians was relatively high.

**Table 4.** The willingness to undergo testing among international immigrants by religion(N=1432^a^).

Variable		Christianity (n=641)	Islam (n=565)	Hinduism (n=73)	Buddhism (n=47)	Others (n=11)	No religion (n=95)
**Have ever tested for STIs^b^/HIV, n (%)**							
	Yes	378 (58.97)	469 (83.01)	58 (79.45)	36 (76.60)	6 (54.55)	65 (68.42)
	No	263 (41.03)	96 (16.99)	15 (20.55)	11 (23.40)	5 (45.45)	30 (31.58)
**Perceived risk of being infected with STIs/HIV, n (%)**							
	Extremely likely	30 (4.68)	18 (3.19)	4 (5.48)	3 (6.38)	2 (18.18)	2 (2.11)
	Very likely	10 (1.56)	20 (3.54)	1 (1.37)	1 (2.13)	0 (0)	5 (5.26)
	Possible	40 (6.24)	41 (7.26)	6 (8.22)	2 (4.26)	1 (9.09)	4 (4.21)
	Unlikely	196 (30.58)	138 (24.42)	16 (21.92)	13 (27.66)	4 (36.36)	33 (34.74)
	Impossible	365 (56.94)	348 (61.59)	46 (63.01)	28 (59.57)	4 (36.36)	5 (53.68)
**Will test for STIs/HIV in the future, n (%)**							
	Refuse	77 (12.01)	158 (27.96)	20 (27.40)	14 (29.79)	1 (9.09)	16 (16.84)
	It doesn’t matter	256 (39.94)	255 (45.13)	35 (47.95)	19 (40.43)	6 (54.55)	45 (47.37)
	I’d like to	308 (48.05)	152 (26.90)	18 (24.66)	14 (29.79)	4 (36.36)	34 (35.79)

a One person was missed in the reporting.

b STIs: sexually transmitted infections.

Table 5 shows that a total of 50 (83.11%) Asians, 399 (60%) sub-Saharan Africans, 20 (45.45%) North Americans, 14 (60.87%) South Americans, 29 (72.50%) Europeans, and 12 (85.71%) North Africans had STIs/HIV test experiences. There were 3 (13.04%) South Americans who believed that they were extremely likely to be infected with STIs/HIV, and 11 (78.57%) North Africans believed that it was impossible for them to be infected with STIs/HIV, which accounted for the largest percentages. As for the willingness to undergo STIs/HIV testing in the future, the willingness of Asians was relatively low and the willingless of sub-Saharan Africans was relatively high.

**Table 5.** The willingness to undergo testing among international immigrants by geography.

Variable		Asia (n=610)	Sub-Saharan Africa (n=665)	North America (n=44)	South America (n=23)	Europe (n=40)	North Africa (n=14)	Others (n=6)
**Have ever tested for STIs** ^**a**^ **/HIV, n (%)**								
	Yes	50 (83.11)	399 (60)	20 (45.45)	14 (60.87)	29 (72.50)	12 (85.71)	4 (66.67)
	No	103 (16.89)	266 (40)	24 (54.55)	9 (39.13)	11 (27.50)	2 (14.29)	2 (33.33)
**Perceived risk of being infected with STIs/HIV, n (%)**								
	Extremely likely	19 (3.11)	30 (4.51)	2 (4.55)	3 (13.04)	2 (5)	0 (0)	0 (0)
	Very likely	22 (3.61)	12 (1.80)	2 (4.55)	0 (0)	0 (0)	0 (0)	0 (0)
	Possible	42 (6.89)	45 (6.77)	1 (2.27)	2 (8.70)	2 (5)	0 (0)	1 (16.67)
	Unlikely	152 (24.92)	204 (30.68)	19 (43.18)	2 (8.70)	14 (35)	3 (21.43)	3 (50)
	Impossible	375 (61.48)	374 (56.24)	20 (45.45)	16 (69.57)	22 (55)	11 (78.57)	2 (33.33)
**Will test for STIs/HIV in the future, n (%)**								
	Refuse	191 (31.31)	63 (9.47)	4 (9.09)	4 (17.39)	8 (20)	2 (14.29)	2 (33.33)
	It doesn’t matter	256 (41.97)	285 (42.86)	20 (45.45)	10 (43.48)	22 (55)	8 (57.14)	2 (33.33)
	I’d like to	163 (26.72)	317 (47.67)	20 (45.45)	9 (39.13)	10 (25)	4 (28.57)	2 (33.33)

a STIs: sexually transmitted infections.

### Religion and Geography Factors Associated With Risky Sexual Behaviors in International Immigrants

The results of binary logistic regression modeling performed to identify factors significantly associated with risky sexual behaviors are shown in Table 6. In multivariate analyses, Muslim (adjusted odds ratio [AOR] 0.453, 95% CI 0.228‐0.897), Hindu (AOR 0.280, 95% CI 0.082‐0.961), and Buddhist (AOR 0.097, 95% CI 0.012‐0.811) immigrants were less likely to report engaging in unprotected sexual behaviors. Moreover, Buddhist (AOR 0.292, 95% CI 0.086‐0.990) were also less likely to have multiple sexual partners. With regard to geography, compared to Asians, South Americans (AOR 2.642, 95% CI 1.034‐6.755), Europeans (AOR 2.310, 95% CI 1.022‐5.221), and North Africans (AOR 3.524, 95% CI 1.104‐11.248) had a higher probability of having multiple sexual partners. shows the multiplicative interaction between religion and geography in relation to risky sexual behaviors, indicating that there was no significant multiplicative interaction. Additionally, with regard to having multiple sexual behaviors, the additive interaction analysis indicated that the relative excess risk due to interaction was –0.731 (–4.957 to 3.495), the attributable proportion due to interaction was –0.526 (–3.732 to 2.680), and the synergy index 0.347 (0.002-63.644). Similarly, with regard to having unprotected sexual behaviors, the relative excess risk due to interaction was 0.197 (-3.320-3.715), the attributable proportion due to interaction was 0.116 (–1.899 to 2.131), and the synergy index was 1.390 (0.002-1041.066). Therefore there was no significant interaction between religion and geography in relation to risky sexual behaviors among international immigrants in this study (Table 7).

**Table 6.** Logistic regression results of the impact of religion and geography on risky sexual behaviors. The italicized *P* values are considered significant.

Variable		Having multiple sexual partners		Having unprotected sexual behaviors	
		AOR^a^ (95% CI)	*P* value	AOR (95% CI)	*P* value
**Religion**					
	Christianity	0.870 (0.510-1.510)	.62	0.801 (0.433-1.483)	.49
	Islam	0.627 (0.346-1.139)	.13	0.453 (0.228-0.897)	*.02*
	Hinduism	0.638 (0.266-1.526)	.31	0.280 (0.082-0.961)	*.04*
	Buddhism	0.292 (0.086-0.990)	*.048*	0.097 (0.012-0.811)	*.03*
	Others	0.810 (0.182-3.604)	.78	0.682 (0.128-3.640)	.66
	No religion	Reference	—^b^	Reference	—
**Geography**					
	Asia	Reference	—	Reference	—
	Sub-Saharan Africa	1.394 (0.897-2.167)	.14	1.625 (0.940-2.811)	.08
	North America	0.751 (0.316-1.783)	.52	0.869 (0.316-2.394)	.79
	South America	3.684 (1.379-9.840)	*.009*	2.369 (0.793-7.073)	.12
	Europe	2.310 (1.022-5.221)	*.04*	2.089 (0.816-5.346)	.12
	North Africa	3.524 (1.104-11.248)	*.03*	2.669 (0.545-13.063)	.23
	Others	1.471 (0.214-10.110)	.70	2.308 (0.314-16.948)	.41

a AOR: adjusted odds ratio.

b Not applicable.

**Table 7.** The multiplicative interaction between religion and geography (depicted by *) in relation to risky sexual behaviors.

Religion*geography	Having multiple sexual partners		Having unprotected sexual behaviors	
	AOR^a^ (95% CI)	*P* value	AOR (95% CI)	*P* value
No religion*sub-Sahara Africa	Reference	—^b^	Reference	—
Christianity*North America	3.147 (0.433-22.851)	.26	2.077 (0.236-18.270)	.51
Christianity*South America	0.758 (0.099-5.814)	.79	0.630 (0.068-5.877)	.69
Christianity*Europe	0.554 (0.074-4.146)	.56	—	.98
Christianity*Asia	1.178 (0.261-5.321)	.83	0.948 (0.171-5.251)	.95
Islam*Europe	2.235 (0.254-19.640)	.47	3.037 (0.312-29.596)	.34
Islam*Asia	0.686 (0.171-2.747)	.59	0.664 (0.132-3.342)	.62

a AOR: adjusted odds ratio.

b Not applicable.

## Discussion

### Principal Findings

This study analyzed aspects of sexual behavior that were potentially influenced by religion and geographic region of origin in international immigrants living in China. Generally, immigrants who identified with a religion were less likely to report engaging in unprotected sex or having multiple sexual partners. Previous studies have also indicated that religion can exert a degree of social control on believers concerning sexual attitudes and behaviors [29]. Religious people tend to have more conservative attitudes toward sex [30]. In this study, Muslims, Hindus, and Buddhists were significantly less likely to engage in unprotected sexual behaviors compared to nonreligious participants (*P*<.05). In a previous study, African-American women reported that religious affiliation was associated with unprotected sex, and increased religiosity predicted a decrease of condomless sex [31]. Another prior study demonstrated that sexual standards among Muslims were reportedly paradoxical, with two opposing attitudes toward sex resulting in the back-and-forth swing in modern society; however, these standards have become increasingly restrictive now [32]. In a study conducted on men who have sex with men in China, Muslims were reportedly less likely to engage in unprotected sex, which aligns with the findings of our study [33]. The majority of the Muslims had STIs/HIV testing experiences, which was consistent with a prior study among Muslim Americans [34]. However, Muslims also had a low willingness to undergo STIs/HIV testing in the future. This may be because Muslims felt reassured after completing initial testing and believed that they were at low risk of contracting STIs/HIV, so their future willingness to undergo testing was relatively reduced. Notably, Buddhists were less likely to have unprotected sexual behaviors and multiple sexual behaviors than nonreligious people. Compared with other religions, Buddhism has less strict rules about sexual behaviors, but Buddhists are also taught to observe four ethical precepts that may limit their sexual behaviors. These precepts state the following: no harming (no killing), no stealing, no sexual misconduct, and no lying [35]. No sexual misconduct means abstaining from deception and betrayal within intimate or committed relationships. This precept aligns with the virtues of fidelity and purity of heart [36]. In a study conducted in Australia, compared with a nonreligious group, Buddhists were significantly more likely to be monogamous and less likely to engage in risky sexual behaviors, which is consistent with the results of our study [29].

In this study, South Americans, Europeans, and North Africans were more likely to have multiple sexual partners and engage in unprotected sexual behaviors compared to Asians. The rate of engagement in unprotected sexual behaviors was highest in immigrants from South America, and the rate of having multiple sexual partners was also highest in immigrants from South America. In a study published in 2020, the prevalence of risky sexual behaviors among undergraduate students in Brazil was high [37]. The number of female sex workers is also high in South America, which may influence sexual attitudes, increasing rates of engagement in commercial sex with multiple partners and having unprotected sex [38,39]. Polygamy still exists in some South American and North African countries [40], and it may have influenced the sexual behaviors of people who migrate from these countries to other places, including acceptance of multiple sexual partners. Notably, global HIV data indicate that African regions had the highest number of people living with HIV in 2022 [41]. In 2022, the incidence rate of HIV in Southeast Asia was 6/100,000, compared to 57/100,000 in the African region, 20/100,000 in the European region, and 18/100,000 in the Americas [41]. In our study, compared with other geographical regions, immigrants from Asian regions had the lowest incidence of having multiple sexual partners and engaging in unprotected sex. This may be strongly influenced by Confucian culture, which compels a large number of Asians to maintain traditional conservative attitudes toward sexual behavior. In many Asian cultures, particularly Muslim communities, the supervision of female and male circumcision also limits the spread of STIs [42].

As a marginal group, immigrants have to experience cross-cultural adaptation. China is a nonreligious country; there may be few religious activities for religious international immigrants. Besides the differences in religious customs, they also face isolation and loneliness in the new living environment by the disruption of the social networks; the relatively poor living space; or the unfamiliarity with the language, the customs, and even the weather, which may result in their high rates of unethical sexual behaviors [43,44]. Moreover, China has a special cultural environment, such as the “condom culture” in terms of safe sex, which in the context of Chinese culture, can be considered as distrust of other parties and is often associated with “nightclubs,” commercial sexual behaviors, and stigmatization [12]. A previous study demonstrated that religious social capital was associated with higher levels of social support among international immigrants, and social support could reduce immigration stress effectively [45]. Additionally, another study in Nigeria [46] indicated that religion was beneficial to the quality of life of people living with HIV. Specifically, intrinsic religiosity, extrinsic religiosity, and positive religious coping had a positive correlation with psychological and physical health as well as the quality of life among people living with HIV. In such situations, religion is beneficial to health-promoting behaviors, as some religious communities are inclined to preach health education, which includes information delivered through faith ministers, religious leaders, and clergy. Furthermore, social support and encouragement from congregants are likely to foster acceptance of undergoing HIV testing [47]. Therefore, it may be a good way to organize religious activities in immigrant communities appropriately.

The reported rates of STIs/HIV testing experience and willingness to take these tests in the future were not high. This may be because many international immigrants are fearful of positive results or are reluctant to disclose private issues (eg, diagnosis of an STI) in a foreign country [48]. However, unawareness of HIV infection may result in severe consequences, contributing to the HIV/AIDS epidemic [49]. Additionally, international immigrants have proven to be risk groups for HIV [21]. Therefore, as an innovation, HIV self-testing should be promoted to increase the uptake and frequency of testing among international immigrants who are missed by existing services [50]. In addition, pre-exposure prophylaxis (PrEP) is widely recognized to reduce the risk of HIV acquisition among HIV-negative people; however, the awareness of PrEP among international immigrants is low [51]. A previous study indicated that language, cost, and medical system are the main barriers to acquiring HIV prevention, such as PrEP, when enhancing the reach of these services to international immigrants [52]. China is still developing the necessary legal regulations, implementation guidance, and standard operating procedures, which are needed for improving the coverage of PrEP. For instance, some researchers indicated that community-driven social media and web-based strategies are effective ways to promote PrEP use among Latino immigrant men [53]. Furthermore, sexual health education is still an effective and feasible way to enhance people’s awareness about the severe consequences of risky sexual behaviors. For example, health workshops could be held regularly in immigrant communities; carrying out STI/HIV-related knowledge contests with prizes may also have noticeable effects.

### Limitations

First, the questionnaire was distributed via the internet, which entails a number of extensively previously described limitations, such as the accuracy of the results. However, it should be noted that web-based surveys also have advantages in numerous contexts, for example, avoidance of potential embarrassment during face-to-face surveys containing sex-related items. Second, it is possible that some international immigrants were compelled by social and cultural norms to hide their sexual behaviors. Third, given the very large numbers of international immigrants now relocating to China, the generalizability of the study results to the entire population may be limited. Fourth, the questionnaire was designed based on previous work and was amended according to our research participants and objectives. Thus, the validity of the questionnaire in the survey needs to be further tested.

### Conclusions

Sex-related behaviors differ among international immigrants with different geographical regions of origin and religions. South Americans and nonreligious people were more likely to engage in risky sexual behaviors, such as having multiple partners and unprotected sexual behaviors. Given that international immigrants face a myriad of sexual health challenges, it is necessary to promote measures, including HIV self-testing, PrEP implementation, and targeted sexual health education to promote the physical and mental health of international immigrants in China.

## Abbreviations

AOR: adjusted odds ratio
PrEP: pre-exposure prophylaxis
STI: sexually transmitted infection
